# HFKG-RFE: An algorithm for heterogeneous federated knowledge graph

**DOI:** 10.1371/journal.pone.0315782

**Published:** 2025-04-29

**Authors:** Chunjuan Li, Hong Zheng, Gang Liu

**Affiliations:** School of Computer Science and Engineering, Changchun University of Technology, Changchun, China; Khulna University, BANGLADESH

## Abstract

Federated learning ensures that data can be trained globally across clients without leaving the local environment, making it suitable for fields involving privacy data such as healthcare and finance. The knowledge graph technology provides a way to express the knowledge of the Internet into a form more similar to the human cognitive world. The training of the knowledge graph embedding model is similar to that of many models, which requires a large amount of data for learning to achieve the purpose of model development. The security of data has always been a focus of public attention, and driven by this situation, knowledge graphs have begun to be combined with federated learning. However, the combination of the two often faces the problem of federated data statistical heterogeneity, which can affect the performance of the training model. Therefore, An Algorithm for Heterogeneous Federated Knowledge Graph (HFKG) is proposed to solve this problem by limiting model drift through comparative learning. In addition, during the training process, it was found that both the server aggregation algorithm and the client knowledge graph embedding model performance can affect the overall performance of the algorithm.Therefore, a new server aggregation algorithm and knowledge graph embedding model RFE are proposed. This paper uses the DDB14, WN18RR, and NELL datasets and two methods of dataset partitioning to construct data heterogeneity scenarios for extensive experiments. The experimental results show a stable improvement, proving the effectiveness of the federated knowledge graph embedding aggregation algorithm HFKG-RFE, the knowledge graph embedding model RFE and the federated knowledge graph relationship embedding aggregation algorithm HFKG-RFE formed by the combination of the two.

## 1 Introduction

The knowledge graph has the ability to organize,manage and understand massive information on the Internet. It’s main idea is to embed triple entities and relationships into a continuous vector space, and express the information in a form closer to the human cognitive world to benefit downstream tasks [1]. In recent years, knowledge graph has been widely applied in fields such as intelligent question answering, intelligent recommendation,and information retrieval [2]. However, knowledge graphs composed of triplets are usually incomplete,and many studies often use embedding existing triplets into continuous vector spaces to predict missing data [3]. The learning process of knowledge graph embedding requires a large amount of data,but data integration faces various problems such as industry competition,privacy and security, and complex administrative procedures. Even achieving data integration between different departments of the same company faces numerous obstacles. In reality, it is almost impossible to integrate data scattered in various places and institutions, or the required cost is huge [4]. Therefore, there are barriers between data sources that are difficult to break down, resulting in the problem of data silos [5]. Federated learning was developed to address the issue of data silos, supporting federated machine learning models that meet user privacy protection, data security, data confidentiality, and government legal requirements [6]. The combination of federated learning and knowledge graphs is considered to ensure data confidentiality and collaborative learning of knowledge graph embedding representations when multiple knowledge graphs are distributed across different clients. The combination of federated learning and knowledge graph often faces the problem of data statistical heterogeneity, which means that the data trained by each participant has the property of nonIID distributed [7]. NonIID data will cause the client to experience model drift during model training [8,9], which will lead to a decrease in the client’s ability to obtain information from other clients through federated learning [10]. In the multiple and interactive training, the performance of the client’s local model cannot be well optimized and improved, resulting in low accuracy of the trained model. Therefore, this paper proposes the algorithm HFKG-RFE to solve the problem of federated data heterogeneity. Two dataset partitioning methods are used on three datasets, DDB14, WN18RR, and NELL, respectively, namely random shuﬄing and splitting of triplets [11] or uneven partitioning of triplets [12]. Different numbers of clients are set up, and extensive experiments are conducted using four knowledge graph embedding models TransE [13], DistMult [14],ComplEx [15]and RotatE [16] to prove that the algorithm HFKG-RFE can effectively solve the problem, and the performance of the algorithm is also improved. In summary, the main contributions of this paper are as follows:

1. Propose an algorithm HFKG to address the problem of data statistical heterogeneity by limiting model drift through contrastive learning, for the scenario of knowledge graph embedding under federated learning settings.
2. Improve the server aggregation algorithm of federated learning to enhance the aggregation effect and achieve the goal of optimizing algorithm performance.
3. Propose a knowledge graph embedding model RFE to improve the local model embedding accuracy of the client, thereby improving and enhancing the overall performance of the federated knowledge graph algorithm.

## 2 Related work

### 2.1 Knowledge graph

Knowledge graph is a technique that uses graph models to describe the relationships between knowledge and modeled things [17–19]. Its main idea is to embed entities and relationships in the knowledge graph into a continuous vector space. In existing works that combine federated learning with knowledge graphs, there are four classic knowledge graph embedding models commonly referenced by local clients: Euclidean embedding TransE [13], which represents relationships and entities in triplets as vectors in the same space for model training. This model shows good performance in large-scale knowledge graphs, but it cannot effectively handle complex relationship problems. Tensor decomposition embedding DistMult [14] limits the bilinear transformation matrix and reduces the number of relationship parameters in the latent variable model, achieving better performance in link prediction tasks. However, its disadvantage is that the model’s combinatorial inference is overly simplified and can only handle symmetric relationships, resulting in weaker performance. Tensor decomposition embedding ComplEx [15] introduces complex vector space, which enables it to capture symmetric and ant symmetric relationships. However, the complex interactions between entities and relationships require a large amount of matrix and vector calculations, resulting in high computational complexity. Euclid embedding RotatE [16] defines each relationship as a rotation from the source entity to the target entity in the complex vector space, thus efficiently and effectively training the model. However, it only has one rotation plane, which also limits the performance improvement during the embedding process.

### 2.2 Federated learning

The common challenge faced by existing federated learning combined with knowledge graphs is the problem of data statistical heterogeneity between different clients, which leads to model drift caused by inconsistent local model training and global model convergence [20–23]. Many algorithms have been proposed to solve this problem, including commonly used federated learning algorithms such as Fedprox and Scaffold. Algorithm Fedprox [24] adds proximal terms to the weights during the client learning process, and relevant experiments have shown that the algorithm Fedprox has better aggregation performance than the algorithm Fedavg. The algorithm scaffold [25] calculates the gradient of local data in the global model or reuses previously calculated gradients, but compared to the algorithm Fedavg, the algorithm Scaffold increases the communication size of each round by approximately twice. In the existing work of combining federated learning with knowledge graphs, client server interaction is often carried out through entities or relationships in dataset triples to train the global model. Research on entity interaction has shown that FedE [26] uses federated learning algorithm Fedavg for entity interaction training models, but the effectiveness is slightly poor when dealing with the problem of data statistical heterogeneity. FKGE [27] uses peer-to-peer joint embedding in federated learning frameworks, which results in high communication costs. FedLU [12] is used for heterogeneous knowledge graph entity embedding learning and cancellation learning, combining backtracking interference and passive decay to aggregate global models. However, the lifecycle of knowledge graphs affects the sustained learning and cancellation learning of algorithms. Research on relational interaction has been conducted by FEDR [11], which has demonstrated through federated model attacks that federated entity embedding is far less secure than federated relationship embedding. Federated knowledge graph entity embedding aggregation can lead to serious privacy breaches, while knowledge graph reconstruction attacks make it difficult to infer entities based on relationships, thus greatly reducing the probability of privacy breaches. In addition, the shared relationship query volume is small and the communication cost is low. However, this algorithm does not consider solving the problem of heterogeneous federated data.

The above work provides corresponding ideas. From the perspective of selecting the interaction relationship between the client and server, which is more secure and has lower communication costs, a federated learning algorithm HFKG is proposed using the embedding comparison idea to solve the problem of data statistical heterogeneity. In addition, a federated learning aggregation algorithm and a knowledge graph embedding model RFE are proposed to improve algorithm performance, combine the improved algorithm to generate the HFKG-RFE algorithm.

## 3 HFKG-RFE algorithm

**Fig 1:**
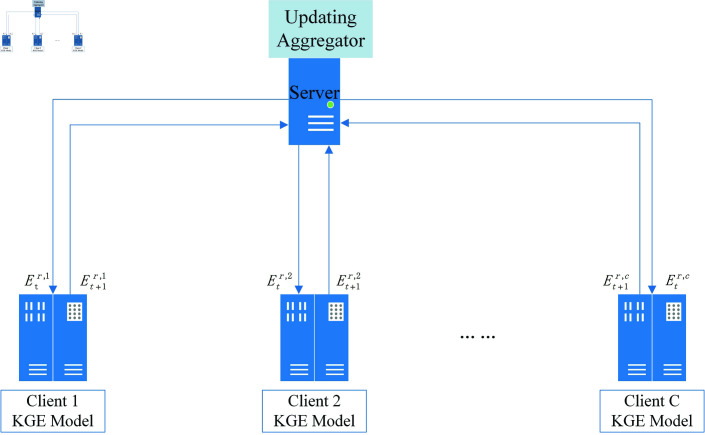
Training process of HFKG-RFE algorithm.

The framework of the HFKG-RFE algorithm is shown in Fig 1, consisting of a server and multiple clients. The client uses its own dataset locally to train the knowledge graph embedding model. The client interacts with the server to obtain a unique relationship matrix. The server aggregates the relationship matrices uploaded by each client and sends them to each client for model optimization and adjustment. Through continuous interactive training, the local knowledge graph embedding model learns more information and achieves optimal model performance.

### 3.1 Federated algorithm HFKG

The overall process of HFKG is as shown in Algorithm 1. Firstly, clients are randomly selected according to the formula F × C , where F is the proportion of clients selected in each round, which is used to select clients according to the proportion of clients. C is the total number of federated learning clients. The server sends the initialized relationship matrix to the selected clients, and the clients perform local knowledge graph embedding model training and update the relationship matrix. Then, the relationship matrix is uploaded to the server for aggregation and updating. Through continuous interaction, each client can learn more knowledge, and the model performance is improved.

**Algorithm 1**.: HFKG-RFE



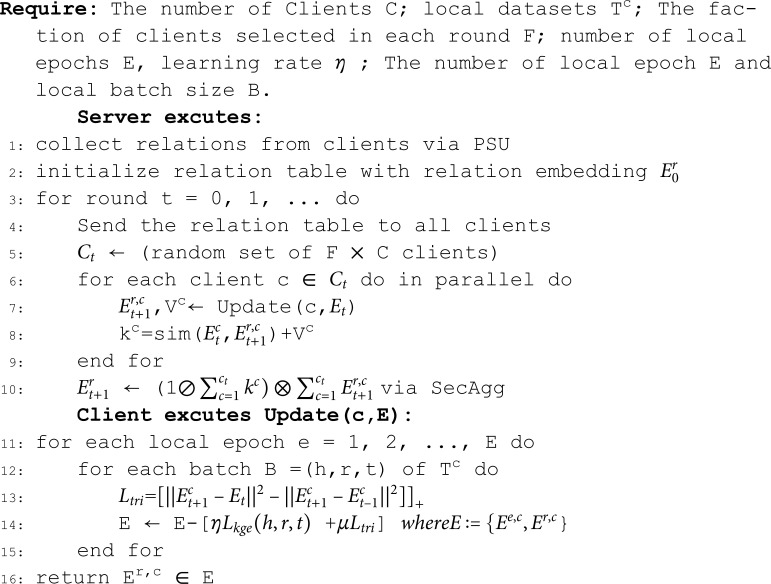



### 3.2 Client update

The four classic knowledge graph embedding models used by federated learning local clients are as follows: TransE [13], DistMult [14], ComplEx [15], RotatE [16]. The scoring function of the knowledge graph embedding benchmark model is shown in Table 1.

**Table 1:** Knowledge graph model rating function.

KGE Model	Score Function	Vector Space
TransE	- | | *h* + *r* − *t* | |	h,r,t ∈ *R*^*d*^
ComplEx	*h*^*T*^ diag(r)t	h,r,t ∈ *R*^*d*^
RotatE	Re(*h*^*T*^ diag(r)t)	h,r,t ∈ *R*^*d*^
DistMult	- | | *h* ∘ *r* − *t* | |	h,r,t ∈ *R*^*d*^

**Fig 2:**
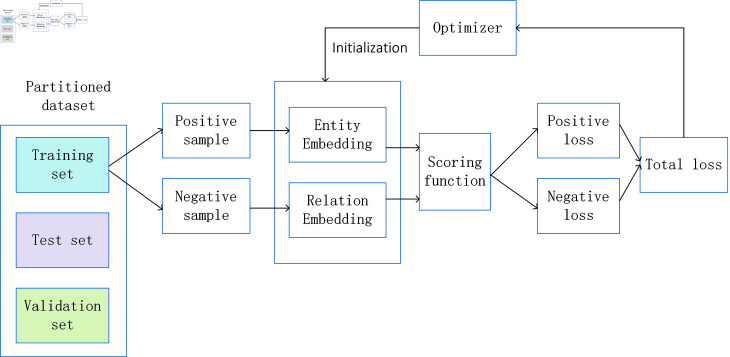
Knowledge graph embedding optimization iteration process.

The federated learning client is based on local data and conducts local training through a set knowledge graph embedding model. The score function is changed by selecting different knowledge graph embedding models. When the client receives a relationship embedding update element embedding *E*^*c*^ issued by the server, for each client C, a selected set of clients is used to adjust the local model and update the shared relationship table. For a triplet (h, r, t) in the knowledge graph on the client, we calculate it using the rating function $f_{r}(h,t)$. The embedding process of the local client knowledge graph is shown in Fig 2, and the loss function is calculated based on the positive and negative samples of the knowledge graph triplet.

The loss function of the client triplets selected according to the client ratio is calculated as Eq (1).


$$\begin{array}{ccc} L(h,r,t)=-log\sigma (f_{r}(h,t)-\gamma )-\overset{N}{\underset{n=1}{\sum }}p(h,r,t^{\prime })log\sigma (\gamma -f_{r}(h,t^{\prime })) & & \end{array}$$ (1)

In the loss function formula, *γ* is the edge of a hyper parameter set, *σ* is the adversarial sampling temperature, and $f_{r}(h,t)$ is the knowledge graph scoring function. (h,r,$t_{i}^{\prime }$) is the negative sample taken corresponding to the knowledge graph triplet (h,r,t), while p(h, r, $t^{\prime }$)is the weight of the corresponding negative sample obtained by calculation, where *α* is the sampling temperature, and the weight is defined as follows:


$$\begin{array}{ccc} p(h,r,t^{\prime })=\frac{exp(\alpha f_{r}(h,t_{j}^{\prime })}{\sum_{i}exp(\alpha f_{r}(h,t_{i}^{\prime })} & & \end{array}$$ (2)

By calculating the numerical gradient of the loss function, combined with an optimizer (such as Adam optimizer), the model parameters are updated based on the gradient information to gradually reduce the loss function, thereby performing local optimization update embedding in the knowledge graph.

**Fig 3:**
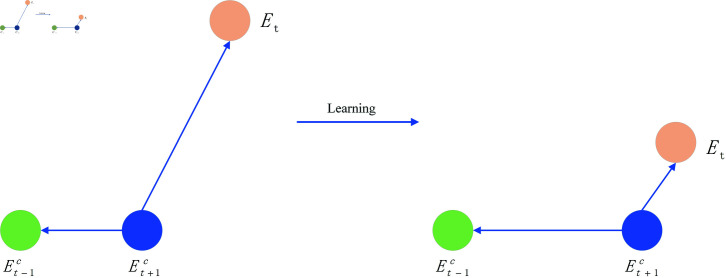
Training process of triplet loss in federated learning.

Inspired by MOON [6], contrastive learning was selected to guide embedding in solving the problem of data statistical heterogeneity. Traditional comparison did not consider the distance relationship between positive and negative samples and anchor points [28–31]. Therefore, the triplet selection method used in FaceNet [32] to solve face recognition problems under different poses and lighting conditions was selected for improvement and applied to federated learning knowledge graph embedding scenarios. The correct sample selection is crucial for model convergence. The target relationship samples required in this paper are the anchor relationship sample matrix $E_{t+1}^{c}$ , the positive relationship sample matrix $E_{t}$ , and the negative relationship sample matrix $E_{t-1}^{c}$ . The selected sample matrices are input into triplet loss to calculate the difference between the local model relationship matrix and the global model relationship matrix to limit model drift and solve the problem of data statistical heterogeneity. Generate roughly aligned matched and unmatched relationship matrix Triplets, and the training process is shown in Fig 3. In each round of training, the federated learning algorithm is promoted to better learn the knowledge graph of each client by minimizing the distance between the relationship matrix $E_{t}$ issued by the server and the latest relationship matrix $E_{t+1}^{c}$ of the client, and maximizing the distance between the latest relationship matrix $E_{t+1}^{c}$ of the client and the relationship matrix $E_{t-1}^{c}$ obtained from the previous round of training.

Specifically, the goal of triplet loss is to minimize the distance between anchor samples and positive samples, and maximize the distance between anchor samples and negative samples. The loss function is defined as:


$$\begin{array}{ccc} L_{tri}=[||E_{t+1}^{c}-E_{t}|{|}^{2}-||E_{t+1}^{c}-E_{t-1}^{c}|{|}^{2}]{]}_{+} & & \end{array}$$ (3)

By minimizing triplet loss, the same class relationship matrix is closer in federated learning algorithms, while different class relationship matrices are more dispersed in federated learning algorithms, thereby improving the ability to handle heterogeneous federated data problems.

### 3.3 Server update

Before the aggregation work on the server, the server obtains the IDs of all unique relationships from the local client and maintains a relationship embedding matrix form. This paper considers improving the aggregation method by considering the correlation between data between the client and server to identify more valuable clients for aggregation. The server receives the initialization relationship embedding matrix form uploaded by each client. In order to improve the aggregation efficiency of the federated server, it is proposed to calculate the cosine similarity between global relationship embedding and client relationship embedding and calculate the relationship existence vector. The specific calculation is shown in (Eq. 4).


$$\begin{array}{ccc} k^{c}=sim(E_{t}^{c},E_{t+1}^{r,c})+V^{c} & & \end{array}$$ (4)

Existence vector calculates the weight of each relationship on all clients, and the cosine similarity value is used to weight the embedding vector of the client relationship, so that the aggregation function not only considers the relationships between clients, but also the relationships between the server and each client. The two are combined to calculate the score of each client in federated learning. By using (Eq. 5), the proportion of each client is calculated by division, and the server aggregation is performed by ratio.


$$\begin{array}{ccc} E_{t+1}^{r}\leftarrow (1⊘\overset{c_{t}}{\underset{c=1}{\sum }}k^{c})⊗\overset{c_{t}}{\underset{c=1}{\sum }}E_{t+1}^{r,c}\;via\;SecAgg & & \end{array}$$ (5)

At the same time, the secure aggregation technology SecAgg [11] proposed by the FEDR algorithm is adopted in the aggregation stage to mask the relationship matrix uploaded by the client, making it impossible for the server to understand the real data, but it does not affect the aggregation effect.

In summary, triplet selection is used to limit model drift to solve the problem of data statistical heterogeneity, and server aggregation methods are improved to enhance the aggregation performance of federated learning on the server side, providing better performance for algorithm training and application.

### 3.4 Knowledge graph embedding model RFE

**Fig 4:**
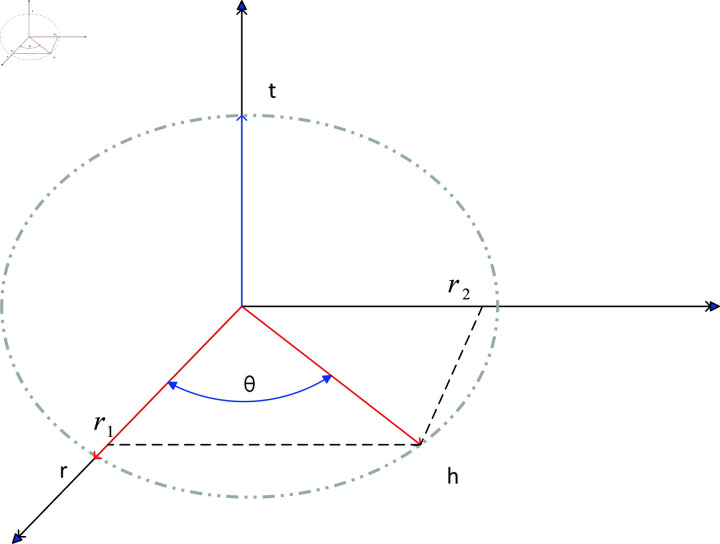
Mapping in three-dimensional space.

**Fig 5:**
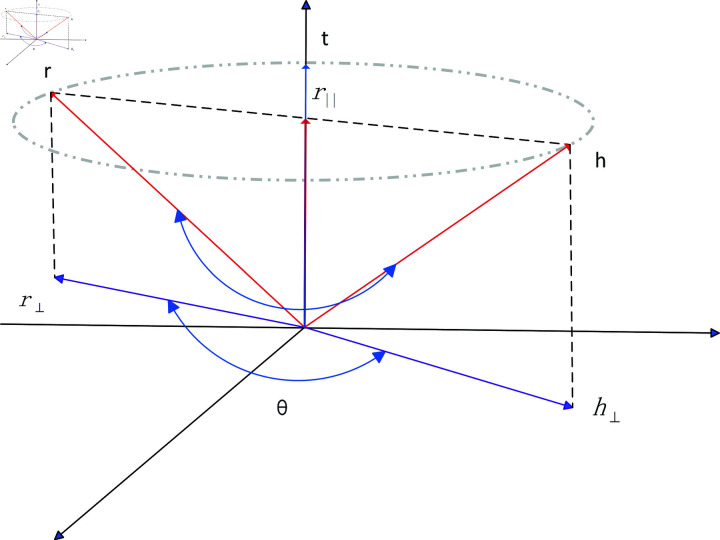
Rotation embedding in three-dimensional space.

In order to improve the aggregation effect on the federated learning server and optimize the performance of the federated algorithm by improving the embedding performance of the federated learning client, a knowledge graph embedding model RFE is proposed. The vector invariance principle of entity and relationship rotation transformation in three-dimensional space is used to achieve vector mapping and splitting of entities and relationships. Combined with the complex calculation formula in complex space, better embedding completion effect is achieved. We project the head and tail entities h and t in three-dimensional space and satisfy the vector calculation formula. Consider the two states in which relationship r exists, Fig 4 shows the state where r and t are perpendicular and Fig 5 shows the state where r and t are not perpendicular.

When the relationship r is perpendicular to the entity t, as shown in Fig 4, for each embedded element, the relationship is mapped, and the entity and relationship satisfy Eq. (6).


$$\begin{array}{ccc} h=r_{1}+r_{2} & & \end{array}$$ (6)

According to the formula for calculating the angle between vectors, obtain Eq. (7) and Eq. (8), where *𝜃* is the rotation angle.


$$\begin{array}{ccc} r_{1}=|r|\cdot cos𝜃\cdot \frac{r}{\left|r\right|}=cos𝜃\cdot r & & \end{array}$$ (7)

$$\begin{array}{ccc} r_{2}=|r|\cdot sin𝜃\cdot \frac{t\cdot r}{|t|\cdot |r|sin<t,r>}=sin𝜃\cdot t\times r & & \end{array}$$ (8)

For each triplet (h, r, t), combined with the complex space calculation of rotating knowledge graph embedding, the distance function of knowledge graph embedding RFE is shown in Eq. (9), where  ∘  is the Hadamard product.


$$\begin{array}{ccc} h=cos𝜃\cdot r+sin𝜃\cdot (t\circ r) & & \end{array}$$ (9)

When the relationship r is not perpendicular to the entity t, as shown in Fig 5, the entity and relationship satisfy Eq. (10).


$$\begin{array}{ccc} h=r_{\left|\right|}+h_{⊥} & & \end{array}$$ (10)

Split the relationship and tail entity into space to obtain (Eq. 11), and then convert it into (Eq. 12) according to the calculation in Eq. (9).


$$\begin{array}{ccc} r_{\left|\right|}=|r|cos<r,t>\cdot t=|r|\frac{r\cdot t}{\left|r|\cdot |t\right|}\cdot t=(r\cdot t)r & & \end{array}$$ (11)

$$\begin{array}{ccc} h_{⊥}=cos\cdot r_{⊥}+sin𝜃\cdot (t\cdot r_{⊥}) & & \end{array}$$ (12)

Therefore, the scoring function calculation formula of the knowledge graph embedding model RFE is combined with the Hadamard product calculation, and the rotating entity and relationship mapping in complex space can be expressed as (Eq. 13), where  ∘  is the Hadamard product.


$$\begin{array}{ccc} f_{r}(h,t)=-h+cos𝜃\cdot r+(1-cos𝜃)\cdot (r\circ t)t+sin𝜃\cdot (t\circ r) & & \end{array}$$ (13)

## 4 Dataset construction

The experiment adopts three different datasets from different fields, the medical database DDB14 [33], the WN18RR [34] dataset, which includes the conceptual semantics and lexical relationships between English words, the NELL [35] dataset, and factual knowledge extracted from hundreds of millions of web pages.

**Fig 6:**
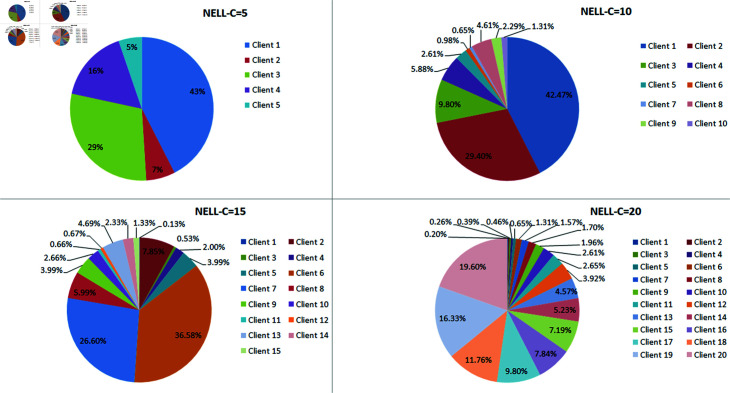
The proportion of triples in each client.

Considering that the knowledge graph dataset is in the form of triplets without labels or features, two methods are adopted for dataset partitioning to form data heterogeneity. The first method is to randomly shuﬄe and split the datasets DDB14 and WN18RR without replacement, so that the triples are not duplicated, and then distribute them to all clients. Random splitting ensures that the data between all clients is heterogeneous [11]. The second method adopts the method of uneven number of triples per client [12]. The dataset is divided into four types, with client numbers C being 5, 10, 15, and 20, and the proportion of triples per client in each type is shown in Fig 6. In addition, the triplet proportions of the training set, validation set, and test set are 0.8, 0.1, and 0.1, respectively.

## 5 Experiment and analysis

In order to evaluate the performance of the HFKG algorithm, the FEDR algorithm with the same shared relationship matrix and the Fedprox algorithm for handling the problem of data statistical heterogeneity were selected for data comparison and analysis. In addition, in order to better conduct comparative analysis of evaluation indicators, the corresponding hyper parameters are set with the same values. The evaluation indicators are: Hits@1, Hits@3, Hits@10 and mean reciprocal rank (MRR).

### 5.1 Federated algorithm HFKG

The experiment evaluates the effectiveness of HFKG algorithm from three perspectives: client, server, and client combined with server.

### 5.2 Client training

The experiment used FEDR as the baseline, and Table 2 shows the link prediction results of dividing the three datasets into C=10 clients. Bold numbers indicate excellent or similar algorithm performance. Compared with FEDR, HFKG generally achieved better or similar evaluation metrics. Taking NELL as an example, the MRR on HFKG increased by 13.02%, 3.99%, 0.45%, and 2.94%, respectively. The method proposed in this paper to solve the problem of data heterogeneity by minimizing the distance between samples is effective. And this method is suitable for situations with a large number of clients.

**Table 2:** Performance of HFKG (triplet selection) on multiple clients divided by different datasets.

KGE	Setting	DDB14-C=10				WN18RR-C=10				NELL-C=10			
		**Hits@1**	**Hits@3**	**Hits@10**	**MRR**	**Hits@1**	**Hits@3**	**Hits@10**	**MRR**	**Hits@1**	**Hits@3**	**Hits@10**	**MRR**
TransE	FEDR	0.2864	0.3171	0.3730	0.3191	0.0071	0.1180	0.1921	0.0737	0.0187	0.1230	0.2757	0.1006
	HFKG(Triplet Selection)	**0.2876**	**0.3193**	**0.3730**	**0.3191**	**0.0161**	**0.1349**	**0.1930**	**0.0829**	**0.0279**	**0.1427**	**0.2913**	**0.1137**
ComplEx	FEDR	0.2915	0.3104	0.3382	0.3097	0.0054	0.0098	0.0188	0.0111	0.0379	0.0756	0.1396	0.0727
	HFKG(Triplet Selection)	**0.2951**	**0.3108**	**0.3391**	**0.3127**	**0.0071**	**0.0125**	**0.0241**	**0.0131**	**0.0398**	**0.0780**	**0.1477**	**0.0756**
RotatE	FEDR	0.2680	0.2937	0.3370	0.2935	0.1126	0.1403	0.1501	0.1296	0.0367	0.0681	0.1292	0.0657
	HFKG(Triplet Selection)	**0.2852**	**0.3038**	**0.3445**	**0.3077**	0.1117	**0.1457**	**0.1582**	**0.1312**	0.0355	0.0665	**0.1307**	**0.0660**
DistMult	FEDR	0.2825	0.2968	0.3220	0.2987	0.1197	0.1278	0.1358	0.1267	0.0346	0.0605	0.1031	0.0578
@@	HFKG(Triplet Selection)	**0.2864**	**0.3014**	**0.3297**	**0.3037**	0.1189	**0.1296**	**0.1367**	**0.1267**	0.0332	**0.0645**	**0.1070**	**0.0595**

### 5.3 Server aggregation

Propose an improved server aggregation algorithm, with the commonly used weighted aggregation algorithm as the baseline in the experiment. Table 3 shows the link prediction results of dividing the three datasets into C=10 client numbers. From the evaluation index data, it can be seen that HFKG performs better. Taking NELL as an example, the MRR on HFKG has increased by 12.92%, 5.09%, 1.83%, and 4.67%, respectively. This indicates that the method proposed in this paper, which weights the relationship embedding vectors of the client by cosine similarity values, is effective and can help the server better aggregate the relationship matrices uploaded by the client.

**Table 3:** Performance of HFKG (aggregation) on multiple clients divided by different datasets.

KGE	Setting		DDB14-C=10			WN18RR-C=10					NELL-C=10		
		Hits@1	Hits@3	Hits@10	MRR	Hits@1	Hits@3	Hits@10	MRR	Hits@1	Hits@3	Hits@10	MRR
TransE	FEDR	0.2864	0.3171	0.3730	0.3191	0.0071	0.1180	0.1921	0.0737	0.0187	0.1230	0.2757	0.1006
	HFKG (Aggregation)	**0.2883**	0.3169	0.3711	**0.3192**	**0.0089**	**0.1269**	**0.2011**	** 0.0781**	**0.0279**	**0.1422**	**0.2915**	**0.1136**
ComplEx	FEDR	0.2915	0.3104	0.3382	0.3097	0.0054	0.0098	0.0188	0.0111	0.0379	0.0756	0.1396	0.0727
	HFKG (Aggregation)	**0.2920**	0.3084	0.3377	0.3094	**0.0071**	**0.0125**	**0.0214**	**0.0126**	**0.0423**	** 0.0790**	**0.1439**	**0.0764**
RotatE	FEDR	0.2680	0.2937	0.3370	0.2935	0.1126	0.1403	0.1501	0.1296	0.0367	0.0681	0.1292	0.0657
	HFKG (Aggregation)	**0.2859**	**0.3043**	**0.3447**	**0.3080**	0.1090	**0.1412**	**0.1528**	0.1286	0.0365	0.0672	**0.1305**	**0.0669**
DistMult	FEDR	0.2825	0.2968	0.3220	0.2987	0.1197	0.1278	0.1358	0.1267	0.0346	0.0605	0.1031	0.0578
	HFKG (Aggregation)	**0.2874**	** 0.2987**	**0.3244**	**0.3023**	0.1180	**0.1323**	**0.1385**	**0.1268**	**0.0346**	**0.0658**	**0.1086**	**0.0605**

### 5.4 Federated algoritnm HFKG

The HFKG algorithm proposed in this paper is generated by combining client improvements and server aggregation improvements. The data in Table 4 shows the link prediction results of dividing the three datasets into C=10 clients. Compared with FEDR, HFKG generally improves the performance of the four evaluation indicators, indicating that the HFKG algorithm performs better than the federated algorithm Fedavg used by FEDR, and the aggregation efficiency has also been improved.

**Table 4:** Performance of HFKG on multiple clients divided by different datasets.

KGE	Setting	DDB14-C=10				WN18RR- C=10				NELL-C=10			
		**Hits@1**	**Hits@3**	**Hits@10**	**MRR**	**Hits@1**	**Hits@3**	**Hits@10**	**MRR**	**Hits@1**	**Hits@3**	**Hits@10**	**MRR**
TransE	FEDR	0.2864	0.3171	0.3730	0.3191	0.0071	0.1180	0.1921	0.0737	0.0187	0.1230	0.2757	0.1006
	HFKG(Ours)	**0.2883**	**0.3181**	0.3716	**0.3193**	**0.0152**	** 0.1367**	**0.1948**	**0.0830**	**0.0276**	**0.1430**	**0.2909**	**0.1134**
ComplEx	FEDR	0.2915	0.3104	0.3382	0.3097	0.0054	0.0098	0.0188	0.0111	0.0379	0.0756	0.1396	0.0727
	HFKG(Ours)	**0.2951**	**0.3108**	**0.3391**	**0.3127**	**0.0071**	**0.0116**	**0.0214**	**0.0123**	**0.0398**	**0.0780**	**0.1477**	**0.0756**
RotatE	FEDR	0.2680	0.2937	0.3370	0.2935	0.1126	0.1403	0.1501	0.1296	0.0367	0.0681	0.1292	0.0657
	HFKG(Ours)	**0.2852**	**0.3038**	**0.3445**	**0.3077**	**0.1117**	**0.1430**	**0.1546**	**0.1300**	0.0353	0.0671	**0.1316**	**0.0658**
DistMult	FEDR	0.2825	0.2968	0.3220	0.2987	0.1197	0.1278	0.1358	0.1267	0.0346	0.0605	0.1031	0.0578
	HFKG(Ours)	**0.2864**	** 0.3014**	**0.3297**	**0.3037**	0.1189	**0.1296**	**0.1367**	**0.1267**	0.0332	** 0.0645**	**0.1070**	**0.0595**

In order to evaluate the performance of the HFKG algorithm in handling the heterogeneity problem of federated data, the datasets DDB14 and WN18RR were divided into C=5,10,15,20 clients. Select the federated learning algorithm Fedprox for data comparison and analysis. The data is shown in Table 5, indicating that the algorithm HFKG has better link prediction performance.

**Table 5:** Results on DDB14 and WN18RR.

Methods		DDB14-C=5				Methods		WN18RR-C=5			
				Hits@1	Hits@3	Hits@10	MRR			Hits@1	Hits@3	Hits@10	MRR
RotatE	Fedprox	0.4303	0.4570	0.5055	0.4571	RotatE	Fedprox	0.1056	0.1554	0.1716	0.1323
	HFKG(Ours)	**0.4314**	0.4588	**0.5071**	**0.4577**		HFKG(Ours)	**0.1056**	**0.1559**	**0.1763**	**0.1339**
**Methods**		**DDB14-C=10**			**Methods**		**WN18RR-C=10**		
		Hits@1	Hits@3	Hits@10	MRR			Hits@1	Hits@3	Hits@10	MRR
ComplEx	Fedprox	0.2941	0.3084	0.3416	0.3117	TransE	Fedprox	0.0071	0.1260	0.1903	0.0749
	HFKG(Ours)	**0.2951**	**0.3108**	**0.3391**	**0.3127**		HFKG(Ours)	**0.0150**	**0.1367**	**0.1948**	**0.0830**
**Methods**		**DDB14-C=15**			**Methods**		**WN18RR-C=15**		
		Hits@1	Hits@3	Hits@10	MRR			Hits@1	Hits@3	Hits@10	MRR
TransE	Fedprox	0.2533	0.2819	0.3460	0.2861	ComplEx	Fedprox	0.0129	0.0172	0.0259	0.0189
	HFKG(Ours)	**0.2539**	**0.2822**	0.3448	**0.2862**		HFKG(Ours)	** 0.0151**	**0.0172**	**0.0302**	**0.0199**
**Methods**		**DDB14-C=20**			**Methods**		**WN18RR-C=20**		
		Hits@1	Hits@3	Hits@10	MRR			Hits@1	Hits@3	Hits@10	MRR
DistMult	Fedprox	0.2378	0.2508	0.2863	0.2574	DistMult	Fedprox	0.1040	0.1280	0.1440	0.1192
	HFKG(Ours)	**0.2397**	0.2500	**0.2878**	**0.2585**		HFKG(Ours)	** 0.1080**	**0.1280**	0.1400	** 0.1208**

In addition, the NELL dataset was divided into different numbers of client triplets, and the client knowledge graph embedding models selected TransE and RotatE for data comparison, as shown in Table 6. Taking MRR as an example, the relative increases compared to the algorithm Fedprox are 8.72%, 6.08%, 5.67%, 3.16% and 4.15%, 2.49%, 3.06%, 2.55%, respectively. Overall, this indicates the ability of HFKG to handle the problem of data statistical heterogeneity.

**Table 6:** Results on NELL.

NELL	Algorithm	TransE				RotatE			
		**Hits@1**	**Hits@3**	**Hits@10**	**MRR**	**Hits@1**	**Hits@3**	**Hits@10**	**MRR**
C=5	Fedprox	0.0254	0.1525	0.3122	0.1170	0.0547	0.1050	0.1957	0.0988
	HFKG(Ours)	**0.0321**	**0.1629**	**0.3281**	**0.1272**	**0.0570**	**0.1099**	**0.2013**	**0.1029**
C=10	Fedprox	0.0230	0.1340	0.2809	0.1069	0.0327	0.0688	0.1297	0.0642
	HFKG(Ours)	**0.0276**	**0.1430**	**0.2909**	**0.1134**	**0.0353**	**0.0671**	**0.1316**	**0.0658**
C=15	Fedprox	0.0260	0.1381	0.2925	0.1128	0.0257	0.0526	0.1067	0.0523
	HFKG(Ours)	**0.0314**	**0.1463**	**0.3027**	**0.1192**	**0.0269**	**0.0532**	**0.1118**	**0.0539**
C=20	Fedprox	0.0265	0.1384	0.2939	0.1138	0.0219	0.0477	0.0962	0.0470
	HFKG(Ours)	**0.0281**	**0.1448**	**0.3027**	**0.1174**	**0.0239**	**0.0483**	**0.0996**	**0.0482**

In the above experimental data comparison, the knowledge graph embedding model trained by HFKG on three datasets achieved good performance improvement, indicating that the proposed HFKG algorithm effectively prevents client model drift and solves the problem of data statistical heterogeneity. However, it was found that Hits@N .The individual data of indicators (N takes 1, 3, 10) did not improve. By calculating the average ranking of the three indicators through MRR, MRR has improved, indicating that the overall proportion of correct triplets in link prediction is high, and the HFKG algorithm is effective.

### 5.5 Knowledge graph RFE

The knowledge graph embedding model RFE combines the FEDR federated algorithm and Fedprox federated algorithm respectively, and compares the model embedding performance with four classic knowledge graph embedding models frequently cited in existing paper work. Table 7 shows that the data is based on the DDB14, WN18RR and NELL datasets, with the average reciprocal rank (MRR) as the metric. The bolded data represents the best knowledge graph embedding performance, indicating that the RFE model has achieved significant results in the federated learning client.

**Table 7:** Combination of RFE embedding model and federated algorithm.

FEDR	DDB14			WN18RR			NELL		
	**C=10**	**C=15**	**C=20**	**C=10**	**C=15**	**C=20**	**C=10**	**C=15**	**C=20**
TransE	0.3191	0.2841	0.2740	0.0737	0.0795	0.0466	0.1006	0.1076	0.1088
ComplEx	0.3097	0.2779	0.2631	0.0111	0.0184	0.0185	0.0727	0.0733	0.0735
RotatE	0.2935	0.2727	0.2614	0.1296	0.1182	0.1210	0.0657	0.0541	0.0461
DistMult	0.2987	0.2707	0.2573	0.1267	0.1153	0.1220	0.0578	0.0555	0.0491
RFE(Ours)	**0.3236**	**0.2948**	**0.2808**	**0.1356**	**0.1468**	**0.1334**	**0.1439**	**0.1407**	**0.1287**
**Fedprox**	**DDB14**			**WN18RR**			**NELL**		
	**C=10**	**C=15**	**C=20**	**C=10**	**C=15**	**C=20**	**C=10**	**C=15**	**C=20**
TransE	0.3197	0.2861	0.2740	0.0749	0.0746	0.0462	0.1069	0.1128	0.1138
ComplEx	0.3117	0.2795	0.2630	0.0217	0.0189	0.0193	0.0936	0.0971	0.0912
RotatE	0.3077	0.2831	0.2707	0.1238	0.1088	0.1108	0.0642	0.0523	0.0470
DistMult	0.3027	0.2713	0.2574	0.1303	0.1186	0.1192	0.0634	0.0618	0.0537
RFE(Ours)	**0.3216**	**0.2879**	**0.2819**	**0.1398**	**0.1445**	**0.1416**	**0.1481**	**0.1474**	**0.1327**

### 5.6 Algorithm HFKG-RFE

Integrate the federated learning algorithm HFKG and the knowledge graph embedding model RFE to obtain the algorithm HFKG-RFE that combines the advantages of both. The algorithm is extensively tested on three datasets: DDB14, WN18RR, and NELL to demonstrate its effectiveness. The bold data in the following Table 8 represents the indicator data with the best results among all algorithm settings.

**Table 8:** Comparison of algorithm performance aesults.

Setting	DDB14			WN18RR			NELL		
	**C=10**	**C=15**	**C=20**	**C=10**	**C=15**	**C=20**	**C=10**	**C=15**	**C=20**
FEDR-TransE	0.3191	0.2841	0.2740	0.0737	0.0795	0.0466	0.1006	0.1076	0.1088
FEDR-ComplEx	0.3097	0.2779	0.2631	0.0111	0.0184	0.0185	0.0727	0.0733	0.0735
FEDR-RotatE	0.2935	0.2727	0.2614	0.1296	0.1182	0.1210	0.0657	0.0541	0.0461
FEDR-DistMult	0.2987	0.2707	0.2573	0.1267	0.1153	0.1220	0.0578	0.0555	0.0491
Fedprox-TransE	0.3197	0.2861	0.2740	0.0749	0.0746	0.0462	0.1069	0.1128	0.1138
Fedprox-ComplEx	0.3117	0.2795	0.2630	0.0217	0.0189	0.0193	0.0936	0.0971	0.0912
Fedprox-RotatE	0.3077	0.2831	0.2707	0.1238	0.1088	0.1108	0.0642	0.0523	0.0470
Fedprox-DistMult	0.3027	0.2713	0.2574	0.1303	0.1186	0.1192	0.0634	0.0618	0.0537
HFKG-RFE(Ours)	**0.3247**	**0.2960**	**0.2821**	**0.1416**	**0.1471**	**0.1432**	**0.1484**	**0.1485**	**0.1387**

Table 8 shows the MRR data indicators and the larger the value of this indicator, the higher the predicted ranking. On all three datasets, HFKG-RFE performed well. Moreover, as the number of clients increased, the data became sparser, and the scene became more complex, the algorithm performance actually surpassed. Compared with other algorithm settings, the link prediction results on heterogeneous datasets were steadily improved, indicating that the algorithm HFKG-RFE has certain effectiveness.

## 6 Conclusion

This paper mainly studies the cross client aggregation relationship matrix to improve the client link prediction ability and improve the knowledge graph while protecting data from leaving the client’s local environment. The HFKG algorithm is the first to introduce the idea of facial recognition and use triplet comparison to address the heterogeneity of federated data between federated learning clients. In addition, algorithms that improve the efficiency of server aggregation ensure that clients with high contribution and value have a larger proportion during aggregation, thereby guaranteeing the aggregation effect. At the same time, the combination of three-dimensional space mapping and the Hadamard product is adopted, and the complex space is used to calculate and generate the knowledge graph embedding model RFE, which achieves significant embedding effects locally on the client side. A large amount of experimental data shows that the proposed algorithm HFKG-RFE has certain effectiveness. Although the addition of Triplet samples increased the computational cost slightly, it achieved the goal of training model accuracy without increasing communication costs. This algorithm will mainly solve the problem of work cost in various calculations and collaborations in the future. In practical applications, clients may simultaneously use multiple models, which may affect overall performance through embedding aggregation. How to ensure the stability of federated learning algorithm performance in this situation and how to better defend against external attacks are the future research directions of this paper.
